# Divergent Trends of Anti-JCPyV Serum Reactivity and Neutralizing Activity in Multiple Sclerosis (MS) Patients during Treatment with Natalizumab

**DOI:** 10.3390/v8050128

**Published:** 2016-05-07

**Authors:** Roberta Antonia Diotti, Ruggero Capra, Lucia Moiola, Valeria Caputo, Nicola De Rossi, Francesca Sangalli, Vittorio Martinelli, Roberto Burioni, Massimo Clementi, Nicasio Mancini

**Affiliations:** 1 Laboratorio di Microbiologia e Virologia, Università “Vita-Salute” San Raffaele, Milan 20132, Italy; diotti.robertaantonia@hsr.it (R.A.D.); caputo.vlr@gmail.com (V.C.); burioni.roberto@hsr.it (R.B.); clementi.massimo@hsr.it (M.C.); 2 Multiple Sclerosis Center, Spedali Civili of Brescia, Brescia 25123, Italy; ruggero.capra@gmail.com (R.C.); ncl.derossi@gmail.com (N.D.R.); 3 Neurological Department, IRCCS Hospital San Raffaele, Milan 20132, Italy; moiola.lucia@hsr.it (L.M.); sangalli.francesca@hsr.it (F.S.); martinelli.vittorio@hsr.it (V.M.); 4 Ospedale San Raffaele, Milan 20132, Italy

**Keywords:** multiple sclerosis, natalizumab, progressive multifocal leukoencephalopathy, humoral response

## Abstract

The association between natalizumab and progressive multifocal leukoencephalopathy (PML) is established, but a reliable clinical risk stratification flow-chart is lacking. New risk factors are needed, such as the possible role of the anti-JC polyomavirus (JCPyV) neutralizing antibody. In this pilot study, we analyzed this parameter during natalizumab treatment. Sequential sera of 38 multiple sclerosis patients during their first year of natalizumab treatment were collected, and grouped according to the number of infusions. For 11 patients, samples were also available after 24 infusions (T24), when progressive multifocal leukoencephalopathy (PML) risk is higher. The reactivity against VP1, the main JCPyV surface protein, and the anti-JCPyV neutralizing activity were evaluated. During the first year, a lack of correlation between anti-JCPyV antibody response and its neutralizing activity was observed: a significant decrease in anti-JCPyV antibody response was observed (*p* = 0.0039), not paralleled by a similar trend in the total anti-JCPyV neutralizing activity (*p* = 0.2239). This lack of correlation was even more evident at T24 when, notwithstanding a significant increase in the anti-JCPyV response (*p* = 0.0097), a further decrease of the neutralizing activity was observed (*p* = 0.0062). This is the first study evidencing, prospectively, the lack of correlation between the anti-JCPyV antibody response and its neutralizing activity during natalizumab treatment.

## 1. Introduction

The association between natalizumab, a humanized monoclonal antibody (mAb) used to treat relapsing-remitting multiple sclerosis (MS) patients, and the development of progressive multifocal leukoencephalopathy (PML) is extensively described [1]. In 2012, a clinical risk stratification flowchart was proposed based on: (1) seropositivity to JC polyomavirus (JCPyV), the PML etiological agent, based on the STRATIFY^®^ test; (2) duration of treatment with natalizumab; and (3) prior use of other immunosuppressor drugs. Although it is currently used, this flowchart has not led to a reduction in PML incidence [2,3]. Recently, the definition of the anti-JCPyV antibody levels measured as “index” has been proposed as an additional stratification marker, but its real clinical utility is still debated [4].

Regarding this stratification flowchart, on the basis of previous studies performed by our group on the antibody response against other persistent viruses [5,6], we proposed to study the anti-JCPyV neutralizing activity and to investigate on its role in progressive multifocal leukoencephalopathy (PML) development [7]. To this purpose, we set up a JCPyV neutralization assay, which revealed the lack of absolute correlation between anti-JCPyV reactivity and neutralizing activity in natalizumab-treated MS patients [8]. Using a similar approach, two recent papers have evaluated the role of neutralizing antibodies for the control of PML-related JCPyV variants, suggesting a possible clinical usefulness of broadly-neutralizing monoclonal antibodies [9,10]. However, to the best of our knowledge, there are no studies in the literature assessing the evolutionary trend of the anti-JCPyV neutralizing the humoral response specifically in natalizumab-treated MS patients.

In this study, we prospectively investigated the trend of the neutralizing anti-JCPyV antibody response in 38 natalizumab-treated MS patients.

## 2. Methods

### 2.1. Serum Samples

Sequential serum samples were collected at three different time-points from 38 patients starting natalizumab therapy at the Multiple Sclerosis Centre of the “Spedali Civili” of Brescia, Italy. Patients’ data are reported in Table 1. The samples were stored in small aliquots at −80 °C until use, and were divided in three groups according to the number of monthly infusions: beginning of therapy (T0), and after 6 (T6) and 12 (T12) months. For 11 patients it was possible to collect samples also after 24 natalizumab infusions (T24), a time-point associated to a dramatic increase in PML risk, according to the currently-followed risk stratification flowchart [2]. Informed consent was obtained from all patients.

### 2.2. JCPyV ELISA

The ELISA assays were performed to test the reactivity of each serum against JCPyV/VP1, by adapting an already described protocol [11]. In brief, ELISA plates were coated with 100 ng/well of a recombinant JCPyV/VP1 (*UniProtKB/Swiss**-**Prot database—*Swiss ID: P03089). The plates were blocked with PBS/1% BSA for 1 h at 37 °C, and then 40 μL of each serum samples (1:200 dilution) were added in duplicate to the wells. After 1 h at 37 °C, the binding was determined using a goat anti-human IgG antibody conjugated to HRP. As a positive control, a single concentration (2 μg/mL) of an anti-JCPyV/VP1 human neutralizing mAb (GRE1) cloned in our lab was used [8]. GRE1 was used also as inter-assay control.

A similar ELISA protocol was followed to detect antibodies against inactivated Herpes simplex virus 1 (HSV1) and 2 (HSV2) viral particles [12].

### 2.3. JCPyV Neutralization Assay

The anti-JCPyV neutralizing activity of each serum was performed using a neutralization assay already described by our group [8,9].

In brief, after complement inactivation at 56 °C for 1 h, each serum (final dilution 1:200) was mixed with medium containing approximately 100 FFU (*fluorescence forming units*) of JCPyV Mad4 strain viral particles (VP1/JCPyV Mad4 strain has the same amino acid sequence of the VP1/JCPyV used in ELISA), and further incubated for 1 h at 37 °C. The mixture was added in triplicate to fibroblast-like COS7 target cells for 2 h at 37 °C, and then it was replaced with fresh medium for 72 h at 37 °C. Cells infected without the addition of serum samples were used as 100% infection control. The number of infected cells was determined using an anti-JCPyV/VP1 commercial murine monoclonal antibody (Ab34756, abcam^®^, Cambridge, UK) for 1 h at 37 °C, which was revealed by a fluorescein isothiocyanate-conjugated anti-murine IgG. The neutralizing activity was expressed as percentage reduction of the number of infected cells in the presence of each serum compared to the infection control. A mini neutralization titer of GRE1 (0.01 μg/Ml ≈ IC_90_, 0.001 ≈ IC_50_ and 0.0001 ≈ IC_10_) and a JCPyV-negative serum were used as positive and negative controls of neutralizing activity, respectively. These controls were used also as inter-assay controls.

A HSV1 neutralization assay was performed following a similar protocol, using kidney epithelial VERO E6 cells as the target cell line [12].

### 2.4. Statistical Analysis

All data were analyzed with a non-parametric test (Friedman test) and Dunn’s multiple comparisons test (as a post test) using Prism 4 software (GraphPad Software Inc., La Jolla, California, USA).

## 3. Results

**Figure 1:**
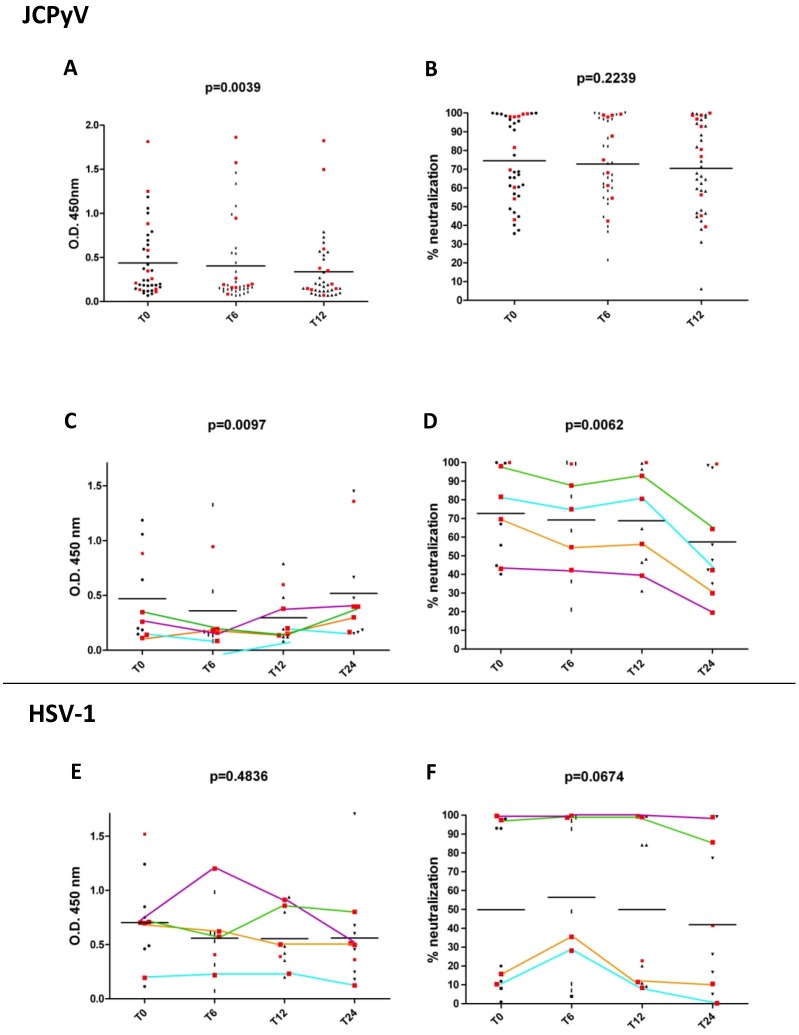
Scatterplots of the serum reactivity and neutralizing activity at different natalizumab infusion time points. The serum reactivity in ELISA against JC polyomavirus (JCPyV)/VP1 (**A**) and the corresponding anti-JCPyV neutralizing activity (**B**) are reported for all the 38 patients at baseline (T0), and after six (T6) and 12 (T12) monthly natalizumab infusions. The same parameters for the 11 patients followed up to 24 infusions (T24) are reported in (**C**) and (**D**); for these patients the serum reactivity in ELISA against HSV-1 viral particles (**E**) and the anti-HSV1 neutralizing activity (**F**) are also reported. The average values at each time point are depicted as black horizontal bars, and the resulting p values calculated by Friedman test are reported. All previously mitoxantrone-treated patients are marked with a red square (■). The four patients with at least a 20% decrease of anti-JCPyV neutralizing activity between T0 and T24 are reported as colored lines; interestingly, the light blue-marked patient developed progressive multifocal leukoencephalopathy (PML) during natalizumab treatment (after the 34^th^ natalizumab infusion). It is important to realize that none of the patients underwent plasma exchange therapy.

During the first year of therapy with natalizumab, a progressive and statistically significant decrease (*p* = 0.0039, with *p*-value < 0.01 for T0 *versus* T12 Figure 1A) of the average serum reactivity against JCPyV/VP1 was observed throughout the different time-points (T0, T6 and T12). Conversely, this significant decrease was not paralleled by a similarly significant decrease of the average anti-JCPyV neutralizing activity (*p* = 0.2239, Figure 1B).

The observed trend changed when the serum samples from 11 patients collected at T24 were analyzed, featuring significant changes both in the average anti-VP1 reactivity and in the anti-JCPyV neutralizing activity (*p* = 0.0097, with *p*-value < 0.05 for T12 *versus* T24 and *p* = 0.0062, with *p*-value < 0.01 for T0 *versus* T24, respectively) (Figure 1C–D). However, and importantly, the observed trends were divergent for the two markers, with the total anti-VP1 reactivity significantly increasing and the anti-JCPyV neutralizing activity significantly decreasing.

In order to evaluate the specificity of the trends observed for the anti-JCPyV antibody response, these 11 sera were studied also in term of the antibody response against Herpes Simplex Virus type 1 (HSV-1), another virus causing a persistent infection. As reported in Figure 1E,F, no significant changes were observed neither in the serum reactivity nor in the neutralizing activity against HSV-1 (*p* = 0.4836 and *p* = 0.0674, respectively).

## 4. Discussion

In this observational study, we reported the differences observed in JCPyV seroreactivity and anti-JCPyV neutralizing activity during treatment with natalizumab. As reported in our previous study [8], this work reveals that, also in prospectively-followed natalizumab-treated patients, the anti-JCPyV antibody response is not strongly correlated to its anti-JCPyV neutralizing activity (Pearson correlation = 0.57366, 0.509618 and 0.527148 for T0, T6, and T12, respectively). In particular, we observed a statistically significant decrease of the anti-JCPyV reactivity at all studied time points, not paralleled by a similar trend of the anti-JCPyV neutralizing activity. Intriguingly, when considering 11 patients, following up to 24 monthly infusions, the decrease of the anti-JCPyV neutralizing activity was strikingly in contrast with a marked and significant increase of the overall response. The limited number of patients in our cohort and the low incidence of PML does not allow any definitive conclusion, but it is interesting to note that the decrease of the anti-JCPyV neutralizing activity was particularly evident in four patients, one of which eventually developed PML (Figure 1D). The specificity of these observations was strengthened by further analysis on the same parameters (reactivity and neutralizing activity) for another persistent virus (HSV-1), against which neither seroreactivity (Figure 1E) nor neutralizing activity (Figure 1F) were significantly modified.

The observed discrepancy between anti-JCPyV reactivity and neutralizing activity has been observed also during the course of other persistent viral infections. The specific humoral response directed against a given pathogen cannot be considered as a whole, but it has to be dissected to its neutralizing and non-neutralizing components (*i.e.*, antibody subpopulation), expressed at different levels during the infection. As an example, this phenomenon was deeply studied by our group in the course of Hepatitis C Virus infection [13,14], during which the different levels of neutralizing and non-neutralizing antibodies have a role in favoring viral immune escape. In particular, the non-neutralizing antibodies have been described to exert an interfering activity against the neutralizing ones [5,15].

Another possible explanation of the observed discrepancies is the emergence of mutated JCPyV strains with novel receptor specificity and altered susceptibility to the serum neutralizing activity. As reported in literature, neurotropic JCPyV strains have two peculiar characteristics: the re-arrangement of a non-coding control region NCCR, and point mutations in VP1, the major structural protein of JCPyV. The Mad4 strain used in this study is neurotropic and it has the same VP1 sequence as the Mad1 strain, which is reported in up to 48% of PML cases [16]. It is, therefore, useful for screening purposes, as the ones reported in this study, and this is also confirmed by the fact that the actual used STRATIFY^®^ screening assay and index calculation are based on Mad1-derived virus-like particles (VLP). However, several other different VP1-mutated neurotropic mutants have been described, and many of them have been reported to escape antibody-mediated neutralization [10]. Some of these strains could be added as VLPs in our neutralization assays to improve its screening effectiveness.

Finally, although obtained during the course of natalizumab treatment, the observed results on the anti-JCPyV serological response cannot be directly correlated to natalizumab, itself. The limited number of patients investigated and the concurrence of several confounding factors, such as previous use of immunosuppressors, may somehow bias the results. For example, 10 out of 38 patients with 12 natalizumab infusions, had been previously treated with mitoxantrone which could have somehow deeply modified the observed B cell response. However, in all mitoxantrone-treated patients’ serum samples were obtained at least nine months after mitoxantrone interruption; moreover, after 30 days of mitoxantrone, all of them featured the same total lymphocytes count as before the treatment. As shown in Figure 1A, all mitoxantrone-treated patients are well distributed along the y-axis at T0, indicating that the previous treatment did not influence the JCPyV reactivity at this time point. Analyzing the data obtained by the patients with 24 natalizumab infusions, we observed that the four patients showing at least a 20% decrease of their anti-JCPyV neutralizing activity between T0 and T24, were all previously treated with mitoxantrone.

However, we think that the observed trend cannot be an aspecific effect of mitoxantrone-induced immunosuppression since a similar trend is not evident in the case of the anti-HSV-1 response.

## 5. Conclusions

This is the first pilot prospective study analyzing the neutralizing humoral response against JCPyV in the course of natalizumab treatment. Notwithstanding its potential biases, we hope that what observed in this study could be the starting point for similar future larger studies. These future studies could also shed some light on the molecular mechanisms leading to the divergent effects observed in the anti-JCVPyV antibody response. Finally, future studies could also allow better evaluation of the potential effectiveness of novel PML prophylactic strategies based on the administration of anti-JCPyV/VP1 neutralizing monoclonal antibodies.

## Figures and Tables

**Table 1:** Patients’ demographic data.

	Overall (*N* = 38)
Sex	
Female (%)	25 (65.8)
Male (%)	13 (34.2)
Age * (years)	
Mean ± SD	34.55 ± 8.18
Median (range)	34 (17–54)
N. of natalizumab infusions (%)	
0–6	38 (100)
7–12	38 (100)
13–24	11 (28.9)
Prior immuno-suppressant use ** (%)	10 (26.3)

*: Age at natalizumab therapy beginning; **: Mitoxantrone.
